# PABPC1 promotes cell proliferation and metastasis in pancreatic adenocarcinoma by regulating COL12A1 expression

**DOI:** 10.1002/iid3.919

**Published:** 2023-07-12

**Authors:** Weijie Yao, Yanrong Yao, Wen He, Chengsi Zhao, Di Liu, Genwang Wang, Zuozheng Wang

**Affiliations:** ^1^ Department of Hepatobiliary Surgery General Hospital of Ningxia Medical University Yinchuan China

**Keywords:** cell metastasis, collagen type XII α1 chain (COL12A1), PABPC1, pancreatic adenocarcinoma

## Abstract

**Background:**

The expression of cytoplasmic poly (A) binding protein‐1 (PABPC1) has been reported in multiple cancer types. This protein is known to modulate cancer progression. However, the effects of PABPC1 expression in pancreatic adenocarcinoma (PAAD) have not been investigated. Here, we investigate the regulatory targets and molecular mechanisms of PABPC1 in PAAD.

**Methods:**

PABPC1 and collagen type XII α1 chain (COL12A1) expression in PAAD and their role in tumor prognosis and tumor stage were investigated using The Cancer Genome Atlas database analysis. After silencing PABPC1, messenger RNA sequencing and Gene Ontology (GO) and Kyoto Encyclopedia of Genes and Genomes (KEGG) analyses were performed. The expression of differentially expressed genes (DEGs), cell viability, apoptosis, and cell migration and invasion were explored using reverse transcription‐quantitative polymerase chain reaction, Cell Counting Kit‐8 assay, flow cytometry assay, and transwell assay, respectively. The relationship between PABPC1 and COL12A1 expression was assessed by Pearson's correlation analysis. The regulatory function of COL12A1 in PABPC1‐affected BXPC3 cell behavior was studied after COL12A1 was overexpressed.

**Results:**

PABPC1 and COL12A1 expression was upregulated in patients with PAAD and was linked to poor prognosis. Four hundred and seventy‐four DEGs were observed in BXPC3 cells after PABPC1 silencing. GO and KEGG analyses revealed that the top 10 DEGs were enriched in cell adhesion pathways. Additionally, PABPC1 silencing inhibited cell viability, migration, and invasion and accelerated apoptosis in BXPC3 cells. PABPC1 silencing increased AZGP1 and ARHGAP30 expression and decreased CAV1 and COL12A1 expression in BXPC3 cells. PABPC1 positively mediated COL12A1 expression, whereas PABPC1 knockdown induced the inhibition of BXPC3 cell proliferation, migration, and invasion.

**Conclusion:**

The results of this study indicate that PABPC1 may function as a tumor promoter in PAAD, accelerating BXPC3 cell proliferation and metastasis by regulating COL12A1 expression.

## Graphical abstract

1. Upregulated cytoplasmic poly (A) binding protein‐1 (PABPC1) is linked to the poor prognosis of pancreatic adenocarcinoma patients; 2. PABPC1 silence regulates the gene expression profile of BXPC3 cells; 3. PABPC1 silence suppresses BXPC3 cells proliferation and metastasis

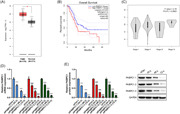

## 1 INTRODUCTION

Pancreatic adenocarcinoma (PAAD) is one of the most malignant tumors of the alimentary tract and is characterized by a high degree of invasion and metastasis.^1^ Early diagnostic strategies for PAAD remain scarce owing to the lack of specific symptoms and biological markers.^2^, ^3^ Currently, surgical excision and chemotherapy are the standard treatments for PAAD; however, achieving a good therapeutic effect in PAAD using these methods is difficult owing to poor prognosis, poor blood supply, and the establishment of an immunosuppressive microenvironment, which may damage the immune system and induce an inflammatory response.^4^, ^5^ Moreover, PAAD shows rapid progression, and more than 80% patients with PAAD are diagnosed with inoperable metastatic tumors.^6^ Therefore, elucidating the mechanism underlying the development and metastasis of PAAD and identifying novel biomarkers and therapeutic targets for disease progression are of great significance.

Poly (A) binding protein (PABP) belongs to a class of highly conserved RNA‐binding proteins (RBPs). PABP is classified into two types: nuclear poly (A) binding protein (PABPN) and cytoplasmic poly (A) binding protein (PABPC).^7^ PABPC1 mediates messenger RNA (mRNA) translation, degradation, and cytoplasmic polyadenylation.^8^, ^9^ Evidence from numerous studies has shown that PABPC1 plays a dual role (tumor promoter or antitumor) in tumor initiation and progression and contributes to tumor cell proliferation, apoptosis, and metastasis and tumor recurrence.^10^, ^11^

The upregulation of PABPC1 has been observed in gastric cancer,^12^ hepatocellular carcinoma (HCC),^13^ and esophageal squamous cell carcinoma (ESCC)^14^ and has been linked to poor prognosis. PABPC1 is a potential prognostic marker. Su et al. reported that PABPC1 prohibited glioblastoma (GBM) cell proliferation, migration, and invasion by promoting the stability of long noncoding RNA brain‐derived neurotrophic factor antisense (lncRNA BDNF‐AS) and inhibiting STAU1‐mediated mRNA degradation.^15^ However, the role of PABPC1 in PAAD has barely been explored.

The abnormal expression of collagen is associated with various pathological processes, especially in malignant tumors.^16^ Collagen type XII alpha 1 chain (COL12A1), encoded by chromosome 6q12‐q13, is a typical collagen‐building molecule involved in collagen cross‐linking in the tumor microenvironment.^17^ At present, the significant role of COL12A1 in multiple cancers is a matter of great concern. Jiang et al. reported the high expression of COL12A1 in gastric cancer cells. This phenomenon may be related to an improvement of the poor overall survival.^18^ Hu et al. reported that COL12A1 expression was high in breast cancer and was related to lymph node metastasis and undesirable overall survival in patients with human epidermal growth factor receptor 2 (HER2)‐enriched breast cancer.^19^ However, the specific function and mechanism of action of COL12A1 in PAAD remain unclear.

In this study, PABPC1 expression in PAAD and its effect on the proliferation, migration, and invasive potential of PAAD cells were investigated using biogenic analysis and cellular experiments. A correlation analysis was conducted between PABPC1 and COL12A1 expression for exploring the underlying mechanism of action of these components. Our findings may provide rare insights into the progression and therapeutic targets for PAAD.

## 2 MATERIALS AND METHODS

### 2.1 The Cancer Genome Atlas (TCGA) database analysis

PAAD data were retrieved from the TCGA database using the Gene Expression Profiling Interactive Analysis tool (GEPIA: http://gepia.cancer-pku.cn/). Using the GEPIA online data analysis website, the PABPC1 and COL12A1 expression levels in 179 PAAD tissues and 171 paracancerous tissues in the TCGA database were analyzed. The correlation between PABPC1 and COL12A1 expression levels and the survival rate or clinical grade of patients with PAAD were analyzed.

### 2.2 RNA extraction and sequencing

TRIZol reagent (No. 15596026; Invitrogen) was used for extracting total RNA from BXPC3 cells. After RNA extraction, the DNA was digested using DNaseI. RNA quality was determined by examining the A260/A280 ratio with the NanodropTM OneC spectrophotometer (Thermo Fisher Scientific). RNA Integrity was confirmed by 1.5% agarose gel electrophoresis. Qubit3.0 with QubitTM RNA Broad Range Assay kit (Life Technologies; Q10210) was used for RNA quantification. Following this, 2 μg of total RNA was used for preparing a stranded RNA sequencing library using the KCTM Stranded mRNA Library Prep Kit for Illumina (Catalog no. DR08402; Wuhan Seqhealth Co., Ltd.) in accordance with the manufacturer's instructions. Polymerase chain reaction (PCR) products with sizes of 200–500 bp were isolated, quantified, and sequenced on a NovaSeq. 6000 Sequencer (Illumina) (PE150 model).

### 2.3 Functional enrichment analysis

Gene Ontology (GO) and Kyoto Encyclopedia of Genes and Genomes (KEGG) analyses were performed to analyze the functional enrichment condition of differentially expressed genes (DEGs). GO and KEGG analyses were performed using the KOBAS 2.0 server.^20^ The top 10 terms with *p* < .05 and counts ≥ 2 were selected.

### 2.4 Cell culture

BXPC3‐PAAD cells were purchased from Promocell and cultivated in Dulbecco's modified Eagle's medium (PM150210, Procell; CHIRON) supplemented with 10% fetal bovine serum (FBS) (SH30084.03; Hyclone). BXPC3 cells were cultured at 37°C in a 5% CO_2_ atmosphere.

### 2.5 Cell transfection

Small interfering RNAs (siRNAs) targeting PABPC1 (siPABPC1‐1, siPABPC1‐2, and siPABPC1‐3) and the matched negative control (siCtrl‐1, siCtrl‐2, and siCtrl‐3) were designed by GenePharma. COL12A1 was overexpressed using pcDNA3.1 vectors (Invitrogen). BXPC3 cells were cultured in a six‐well plate. Thereafter, cell transfection was performed using Lipofectamine^TM^ RNAiMAX Transfection Reagent (#13778150; Invitrogen). The transfection efficiency was measured using reverse transcription‐quantitative polymerase chain reaction (RT‐qPCR) and a western blot analysis assay at 24, 48, and 72 h.

### 2.6 Cell viability assay

Cell Counting Kit‐8 (CCK‐8, CK04; Dojindo) was used for assessing cell viability. After transfection with siPABPC1 and/or COL12A1 vectors, BXPC3 cells were collected and used to prepare a cell suspension. Following this, 100 μL of the BXPC3 cell suspension was added into 96‐well plates and co‐cultured with 10 μL of CCK‐8 solution for 24, 48, and 72 h. Absorbance was measured at 450 nm in a microplate reader (REAGEN).

### 2.7 Cell cycle assay

A Cell Cycle and Apoptosis Analysis Kit (Beyotime) was used for cell cycle analysis. The procedure was performed as directed in the instruction manual provided with the kit. The transfected BXPC3 cells with siPABPC1 and siCtrl were added to 1 mL of precooled 70% ethanol for immobilization for 4 h at 4°C. Following this, BXPC3 cells were stained with 25 μL of propidium iodide (PI) for 30 min in a 37°C water bath in the dark. Flow cytometry (NovoCyte; ACEA Biosciences, Inc.) was used for cell cycle analysis.

### 2.8 Cell apoptosis assay

Flow cytometry was conducted to assess cell apoptosis. Briefly, after transfection with siPABPC1 and siCtrl, the transfected BXPC3 cells were harvested and fixed overnight with 75% ethanol at 4°C. After washing with phosphate‐buffered saline (PBS) three times, 5 μL of PI and 5 μL of fluorescein isothiocyanate Annexin V (#40302ES60; Yesen) were added into the cell plates and kept for 15 min in the dark. Apoptotic BXPC3 cells were analyzed using flow cytometry (NovoCyte).

### 2.9 Cell migration and invasion assay

A transwell assay was conducted using Millicell cell culture inserts (24 well inserts, 8 μm pore size; Corning Incorporated) based on the manufacturer's instructions. For detecting cell migration, 4 × 10^4^ cells in 200 μL of serum‐free medium were added into the base of the inserts, and 500 μL of RPMI‐1640 medium supplemented with 10% FBS was added into the lower chambers. Subsequently, 0.5% crystal violet solution (Beyotime) was used for cell staining. For assessing cell invasion, the inserts were coded with Matrigel (BD Biosciences) for 4 h. The other steps followed were the same as those used in the cell migration assay. Cells in five random fields were counted in each well under an inverted microscope (IX71; Olympus).

### 2.10 RT‐qPCR

After transfection with siPABPC1 (#1, #2, and #3) and siCtrl (#1, #2, and #3), total RNA from transfected BXPC3 cells was extracted using TRIzol® reagent (#10606ES60; Yesen). A HiScript II 1st Strand cDNA Synthesis Kit (+gDNA wiper) (#R212‐01/02; Vazyme) was used for reverse transcription. Subsequently, RT‑qPCR was performed to assess the expression of PABPC1, zinc‐α2‐glycoprotein (AZGP1), Rho GTPase activating protein 30 (ARHGAP30), Caveolin‐1 (CAV1), and COL12A1 using an Applied Biosystems QuantStudio 6 Flex (Applied Biosystems) and Hieff® qPCR SYBR Green Master Mix (Low Rox) (#11202ES08; Yesen). The related primers were as follows: PABPC1, forward: 5′‐ATC CCA CAG ACT CAG AAC CG‐3′, reverse: 5′‐ACA TGA CTC GTG GAA CCT GT‐3′; AZGP1, forward: 5′‐GGA AGC AGG ACA GCC AAC TT‐3′, reverse: 5′‐TTA TTC TCG ATC TCA CAA CCA AAC‐3′; ARHGAP30, forward: 5′‐GGA GGT CAG CAA GGA ACG G‐3′, reverse: 5′‐CAG AGT GGA AGC TAG ACG CAT G‐3′; CAV1, forward: 5′‐GCA ACA TCT ACA AGC CCA‐3′, reverse: 5′‐ CTT CAA AGT CAA TCT TGA CCA C‐3′; COL12A1, forward: 5′‑CCA CAG GTT CAA GAG GTC CC‑3′, reverse: 5′‑TGT GTT AGC CGG AAC CTG GA‑3′; Glyceraldehyde‐3‐phosphate dehydrogenase (GAPDH), forward: 5′‑TCA AGA AGG TGG TGA AGC AGG‑3′, reverse: 5′‑TCA AAG GTG GAG GAG TGG GT‑3′. The mRNA expression levels were normalized to that of GAPDH, which was computed using the $2^{-\Delta \Delta C_{t}}$ method.

### 2.11 Western blot analysis

After transfection, total protein samples were extracted using RIPA lysis Buffer (P0013B; Beyotime). Subsequently, a BCA kit (P0009; Beyotime) was used for determining the concentrations of protein samples. After sodium dodecyl‐sulfate polyacrylamide gel electrophoresis and the electrotransfer of proteins onto poly (vinylidene fluoride) membranes, the membranes were blocked with 5% defatted milk (Yili) for 1 h. The following primary antibodies were used for western blot analysis: anti‐PABPC1 (A14872, 1:1000; Abclonal), anti‐COL12A1 (ab121304, 1:2000; Abcam), and anti‐GAPDH (ATPA00013Rb, 1:5000; ATA). The corresponding secondary antibodies (ab6721, 1:2000; Abcam) were co‐cultivated for 1 h at room temperature. Electrochemiluminescence western blot reagents (K‐12043‐D10; Juneng Yitong Biological Co., Ltd.) were used to visualize the protein blots.

### 2.12 Statistical analysis

SPSS 12.0 software (SPSS Inc.) was used for statistical analyses. The results are presented in terms of mean values ± standard deviation. Principal component analysis (PCA) was used to analyze the differences between the values in the siPABPC1 group and siCrtl group. The Kaplan–Meier (KM) plotter method and Pearson's *χ* ^2^ test were used to determine the overall survival and relevance of PABPC1 and COL12A1 expression. One‐way analysis of variance following Tukey's post hoc test was used for multigroup comparisons. *p* < .05 was considered to be statistically significant.

## 3 RESULTS

### 3.1 PABPC1 expression is upregulated and associated with the poor prognosis of patients with PAAD

**Figure 1:**
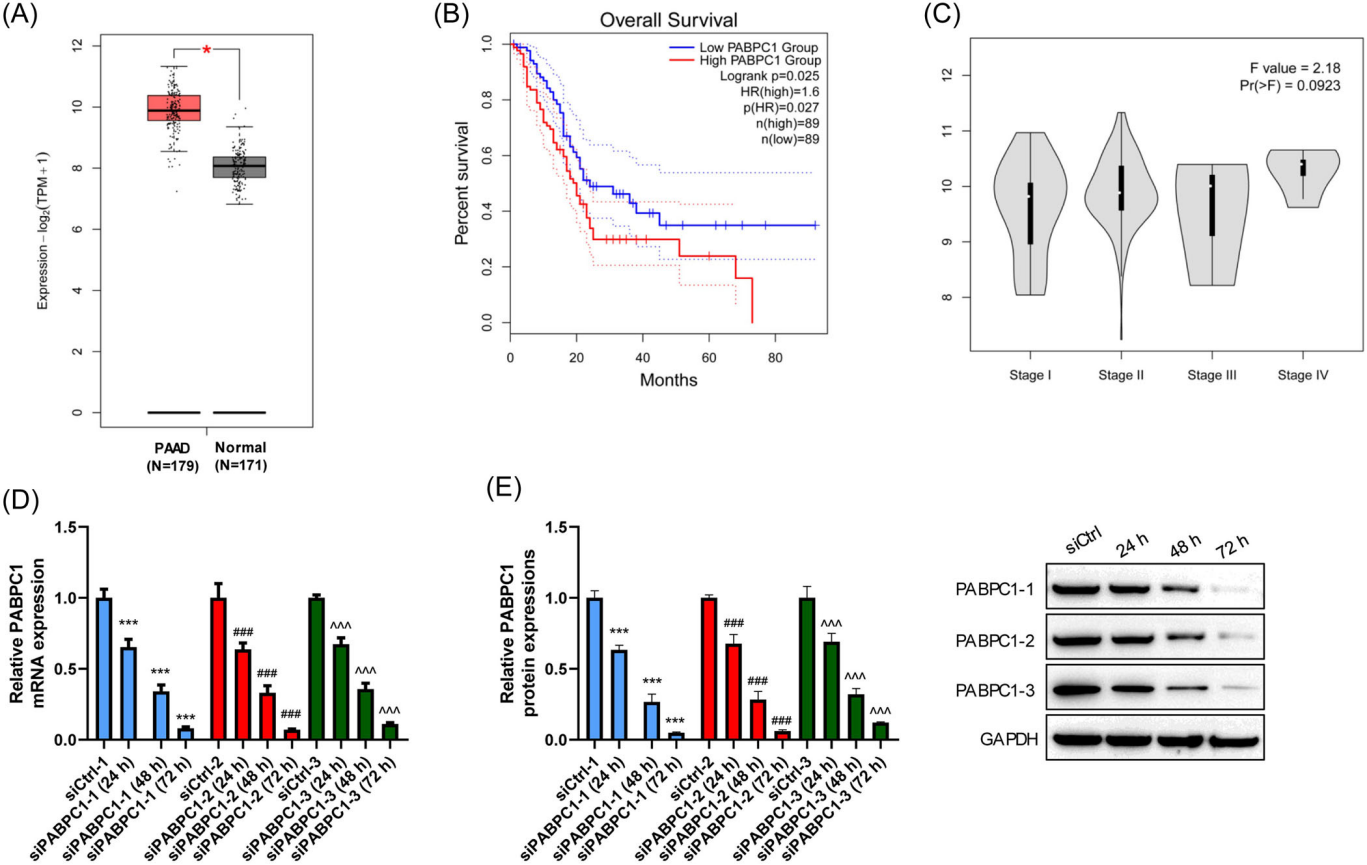
Upregulated cytoplasmic poly (A) binding protein‐1 (PABPC1) in *pancreatic* adenocarcinoma (PAAD) patients is linked to poor prognosis. (A) Based on The Cancer Genome Atlas database, PABPC1 expression level in PAAD patients (*N* = 179) compared with normal subjects (*N* = 171) were assessed. (B) The relevance between PABPC1 and overall survival was researched via Kaplan–Meier plotter analysis (*n* [high] = 89; *n* [low] = 89). (C) Expression of PABPC1 in PAAD patients (*N* = 179) at stage I, II, III, IV was analyzed. After transfection with PABPC1 silenced vectors (siPABPC1) and negative controls (siCrtl), (D) the messenger RNA and (E) protein levels of PABPC1 in BXPC3 cells at 0, 24, 48, and 72 h time points were explored via reverse transcription‐quantitative polymerase chain reaction and Western blot assays. These experiments were repeated for three times and analyzed via SPSS 12.0 software using analysis of variance along with Tukey's post hoc test. *p < .05 versus Normal, ***p < .001 versus siCtrl−1, ^###^ *p* < .001 versus siCtrl−2, ^^^^^ *p* < .001 versus siCtrl−3.

Based on GEPIA2 with the TCGA database, PABPC1 was found to be observably upregulated in patients with PAAD (*N* = 179) compared with that in normal subjects (*N* = 171) (*p* < .05, Figure 1A). Moreover, this was associated with a shorter overall survival time, hinting a poor prognosis (*p* < .05, Figure 1B). At the different phases of clinicopathological staging, we observed that PABPC1 expression in patients with PAAD with a high TNM stage (stage IV) was marginally enhanced compared with that in the patients with a low tumor stage (stage I, II, and III) (Figure 1C). For further evaluation of the effect of PABPC1, silencing vectors (siPABPC1‐1, siPABPC1‐2, and siPABPC1‐3) and negative controls (siCtrl‐1, siCtrl‐2, and siCtrl‐3) were structured and transfected into BXPC3 cells. As depicted in Figure 1D,E, PABPC1 expression at the mRNA and protein levels at 24, 48, and 72 h had declined significantly in BXPC3 cells upon PABPC1 silencing vector transfection. This indicated that the transfection was successful (*p* < .001). The findings confirmed that PABPC1 was abnormally expressed in patients with PAAD, which occurs at an advanced tumor stage and is linked with poor prognosis.

### 3.2 The silencing of PABPC1 affects the gene expression profile of BXPC3 cells

**Figure 2:**
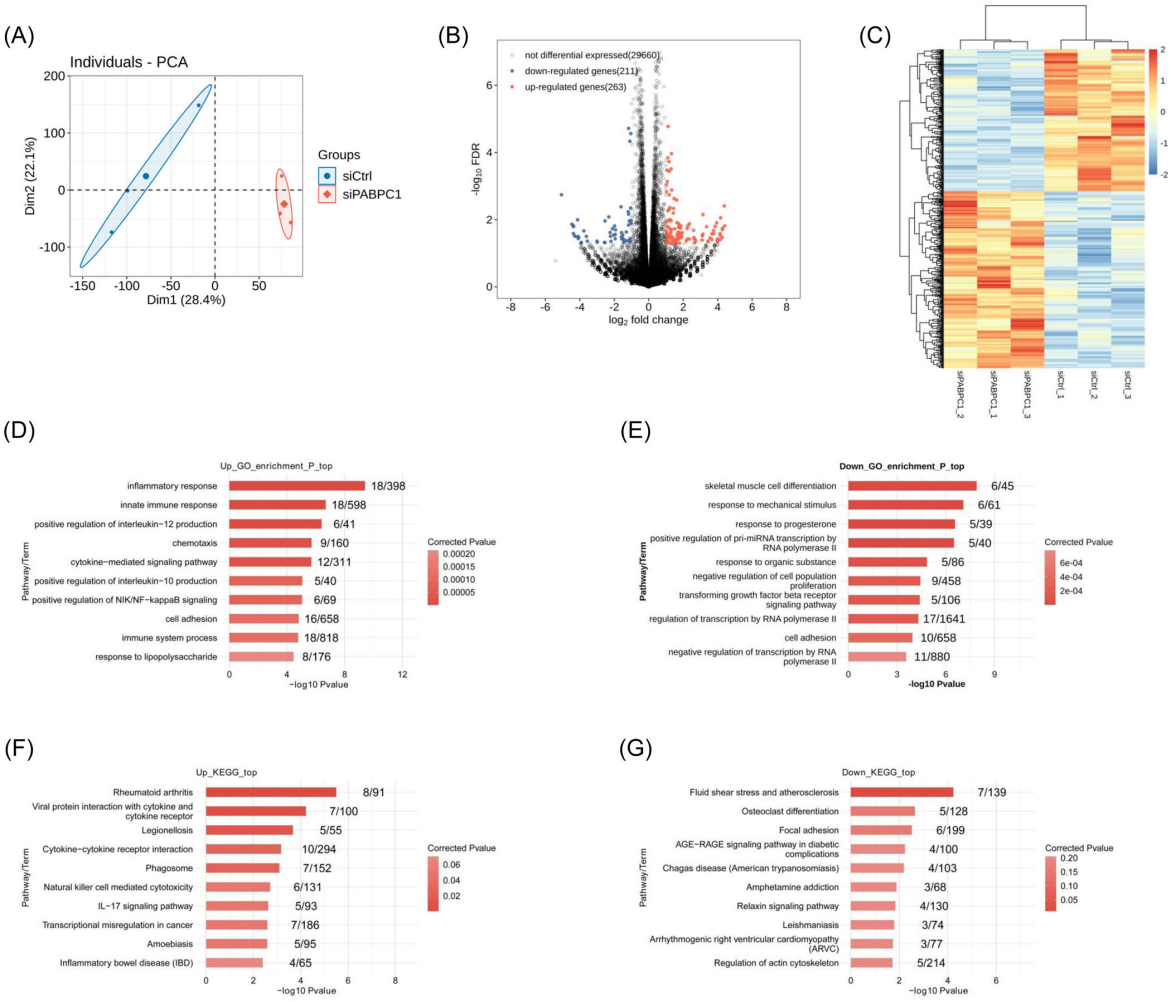
Cytoplasmic poly (A) binding protein‐1 (PABPC1) silence affects the gene expression profile of BXPC3 cells. After transfection with PABPC1 silenced vectors (siPABPC1) and negative controls (siCrtl) for 48 h in BXPC3 cells, (A) The significant differences between siPABPC1 group and siCrtl group was analyzed by principal component analysis (PCA) based on fragments per kilo base per million mapped reads value. (B) The differentially expressed genes (DEGs) between siPABPC1 group and siCrtl group were showcased by volcanic distribution map (the downregulated genes: *n* = 211; the upregulated genes: *n* = 263). *p* < .05 and fold change (FC) ≥2 or ≤1/2. (C) The levels of DEGs were presented by hierarchical cluster analysis (the number of differentially expressed genes was 474). (D–G) The enrichment of DEGs (*n* = 474) was determined by Gene Ontology (GO) analysis and Kyoto Encyclopedia of Genes and Genomes (KEGG) pathway analysis.

After the transfection of BXPC3 cells with PABPC1 silencing vectors and negative controls, RNA‐Seq was performed to gain a comprehensive understanding of the variations in gene expression profiles following PABPC1 downregulation. As shown in Figure 2A, PCA indicated significant differences between the siPABPC1 group and the siCrtl group. The volcano map of variational gene distribution and the results of hierarchical clustering indicated that the number of DEGs in siPABPC1‐transfected BXPC3 cells reached 474, among which 263 genes were upregulated and 211 genes were downregulated (Figure 2B,C). Therefore, PABPC1 silencing may affect the abnormal expression of several genes in PAAD cells.

To elucidate the function of abnormally expressed genes after PABPC1 silencing, we used the GO database to perform functional clustering analysis on these upregulated and downregulated genes. The results indicated that the top 10 upregulated genes in BXPC3 cells with PABPC1 silencing were primarily enriched in cell adhesion (Figure 2D). Additionally, the top 10 downregulated genes in BXPC3 cells with PABPC1 silencing were also associated with the cell adhesion pathway (Figure 2E). Furthermore, KEGG pathway analysis indicated that upregulated genes were markedly enriched in conserved cancer‐correlated pathways, such as cytokine‐cytokine receptor interaction, phagosome, and interleukin‐17 (IL‐17) signaling pathways (Figure 2F). Moreover, the downregulated genes were clearly associated with focal adhesion and relaxin signaling (Figure 2G). These outcomes indicated that PABPC1 silencing contributed to the regulation of gene expression profiles in PAAD cells.

### 3.3 PABPC1 silencing inhibits BXPC3 cells proliferation and metastasis

**Figure 3:**
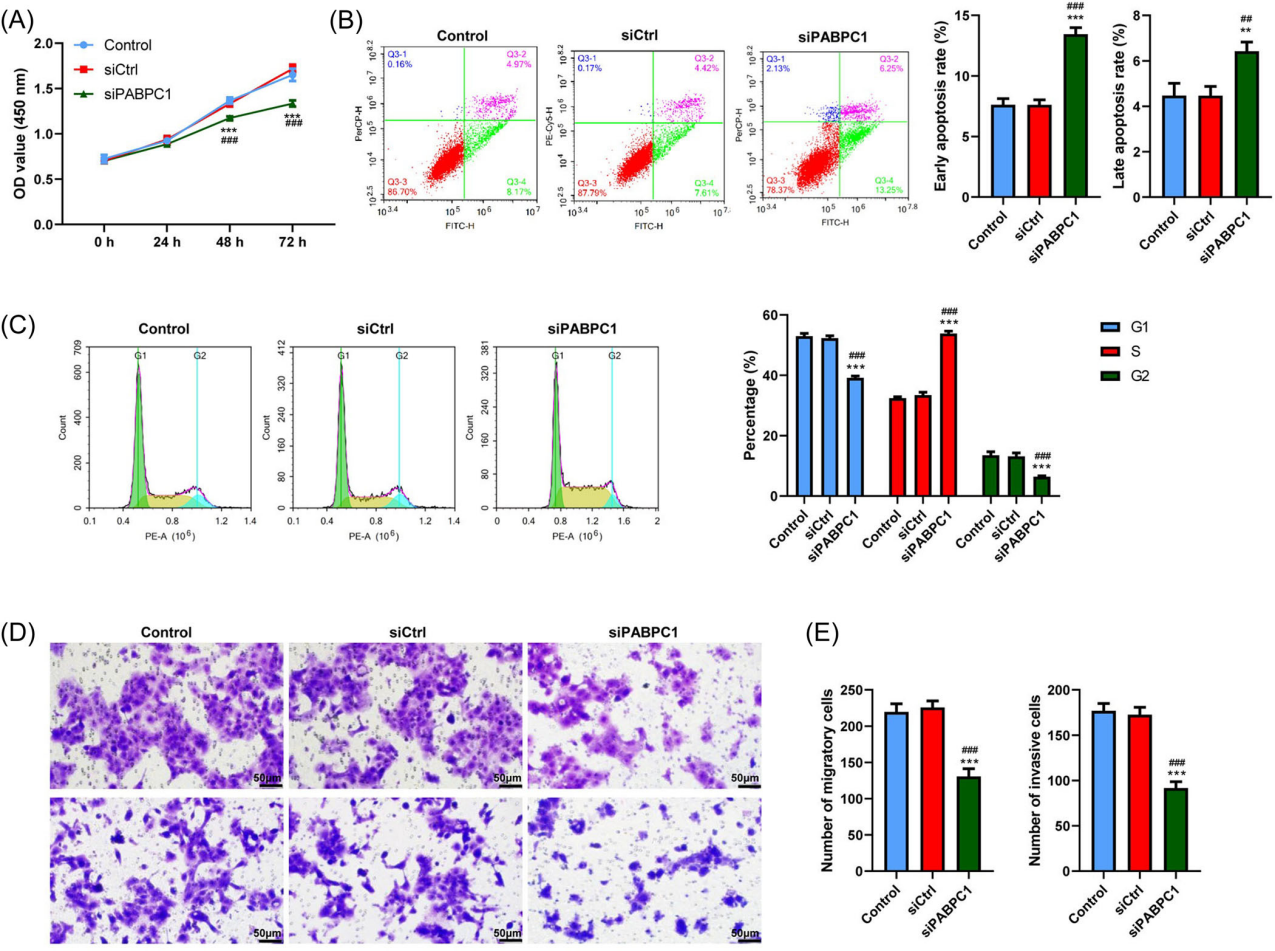
Cytoplasmic poly (A) binding protein‐1 (PABPC1) silence inhibits BXPC3 cells growth and metastasis. The vectors of PABPC1 silenced vectors (siPABPC1) and negative controls (siCrtl) were transfected into BXPC3 cells, (A) Cell viability at the different time points (0, 24, 48, and 72 h) was analyzed via Cell Counting Kit‐8 assay. (B) BXPC3 apoptotic cells and (C) Cell cycle was estimated by flow cytometry assay. (D and E) The migration and invasion of BXPC3 cells were studied by transwell assay (magnification = ×200, Scale bar = 50 μM). These experiments were repeated for three times and analyzed via SPSS 12.0 software using analysis of variance along with Tukey's post hoc test. ***p < .001 versus Control, ^###^ *p* < .001 versus siCtrl.

Next, the functions and mechanisms of action of PABPC1 in the biological behavior of BXPC3 cells were investigated using CCK‐8, flow cytometry, and transwell assays. As shown in Figure 3A, the vitality of BXPC3 cells was significantly suppressed upon PABPC1 silencing (*p* < .001). Meanwhile, the early apoptotic rates of BXPC3 cells were observably increased upon PABPC1 silencing (*p* < .001, Figure 3B). Moreover, the late apoptotic rates of BXPC3 cells were also clearly decreased upon PABPC1 silencing (*p* < .01, Figure 3B). Additionally, PABPC1 silencing triggered BXPC3 cells apoptosis through the induction of cell cycle arrest in the S phase (*p* < .001, Figure 3C). More significantly, the migration and invasion potential of BXPC3 cells was restrained in the siPABPC1 group compared with that in the control or siCtrl group (*p* < .001, Figure 3D,E). These consequences indicated that PABPC1 silencing could inhibit BXPC3 cells viability, migration, and invasion while triggering cells apoptosis.

### 3.4 PABPC1 silencing downregulates COL12A1 expression

**Figure 4:**
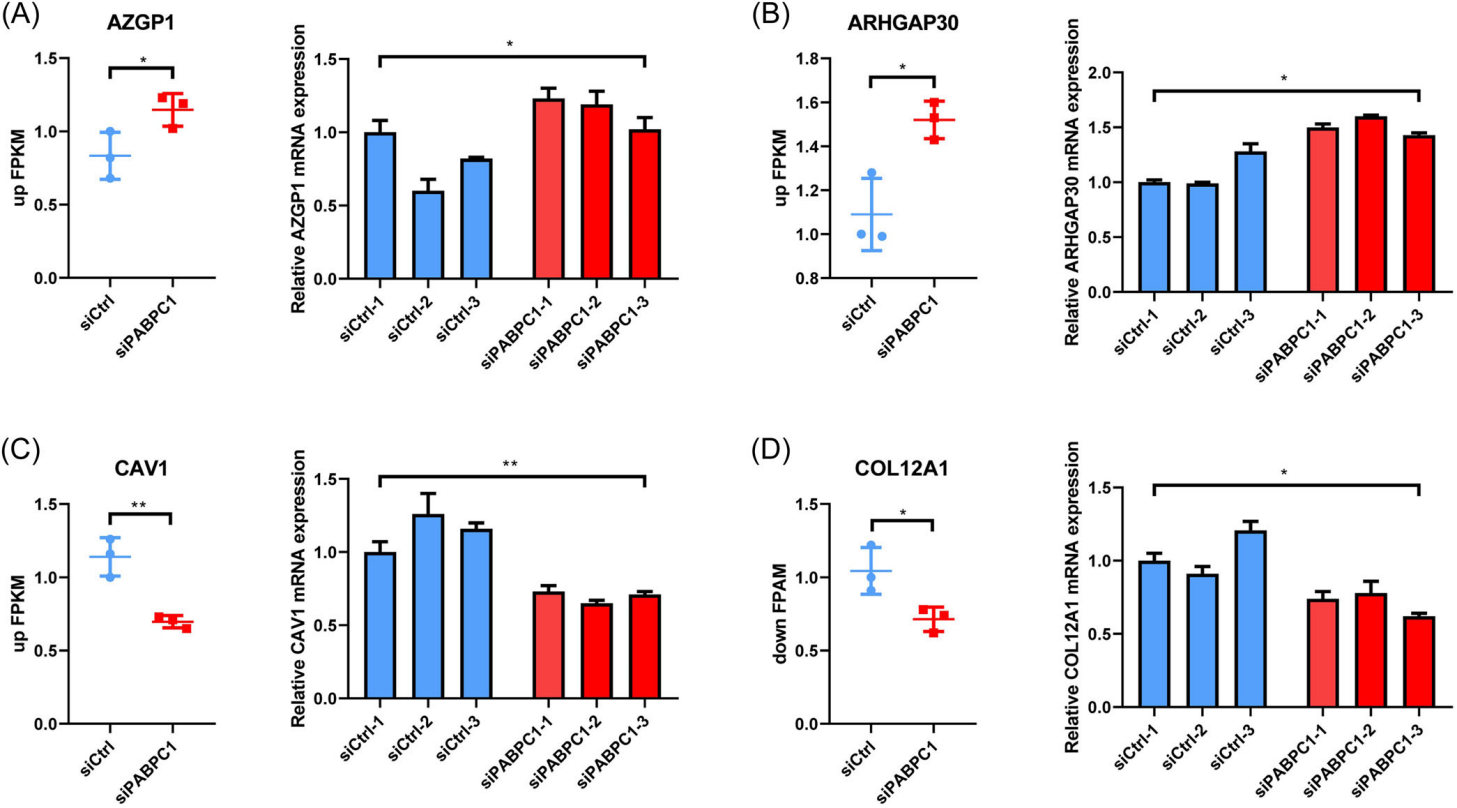
Cytoplasmic poly (A) binding protein‐1 (PABPC1) regulates the expression of correlated genes in BXPC3 cells. After transfection with PABPC1 silenced vectors (siPABPC1) (#1, #2 and #3) and negative controls (siCrtl) (#1, #2 and #3) vectors in BXPC3 cells for 48 h, the differential gene expression levels of (A) zinc‐α2‐glycoprotein (AZGP1), (B) Rho GTPase activating protein 30 (ARHGAP30), (C) Caveolin‐1 (CAV1), and (D) collagen type XII α1 chain (COL12A1) were examined with the aid of reverse transcription‐quantitative polymerase chain reaction assay. These experiments were repeated for three times and analyzed via SPSS 12.0 software using analysis of variance along with Tukey's post hoc test. *p < .05, **p < .01 versus siCtrl.

**Figure 5:**
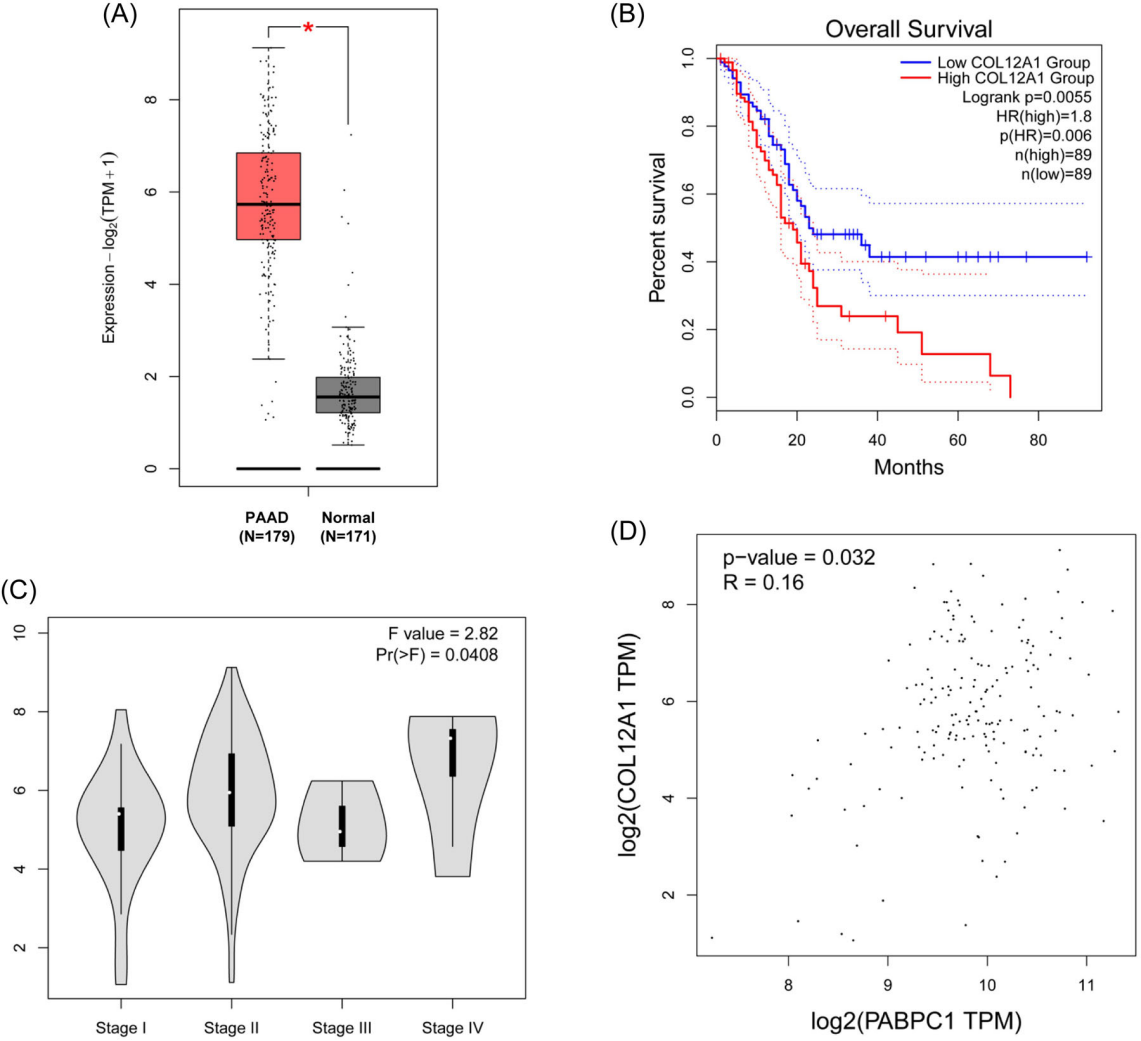
Collagen type XII α1 chain (COL12A1) is upregulated in pancreatic adenocarcinoma (PAAD) patients and is linked to poor prognosis. (A) According to The Cancer Genome Atlas database, COL12A1 expression in PAAD patients (*N* = 179) compared with normal subjects (*N* = 171) was analyzed. (B) The relevance between COL12A1 and overall survival was evaluated via Kaplan–Meier plotter analysis (*n* [high] = 89; *n* [low] = 89). (C) Expression of COL12A1 in PAAD patients (*N* = 179) at stage I, II, III, IV was analyzed. (D) The relationship between COL12A1 and cytoplasmic poly (A) binding protein‐1 (PABPC1) in PAAD samples (*N* = 179) was determined by Pearson's correlation analysis.

Based on the abovementioned research findings, we explored the expression patterns of cell adhesion‐associated genes in BXPC3 cells through RT‐qPCR. As shown in Figure 4A,B, we noticed that PABPC1 silencing upregulated AZGP1 and ARHGAP30 expression levels in BXPC3 cells (*p* < .05). On the contrary, the mRNA levels of CAV1 and COL12A1 markedly declined in siPABPC1‐transfected cells compared with those in siCtrl‐transfected cells (*p* < .05, Figure 4C,D). Following this, we further studied the correlation between COL12A1 expression and the survival of patients with PAAD based on data from the TCGA database. The results indicated that COL12A1 expression was enhanced in 179 patients with PAAD compared with that in 171 normal participants (*p* < .05, Figure 5A). Moreover, high COL12A1 expression was linked to the low survival percentage of patients with PAAD (*p* < .01, Figure 5B). Additionally, we observed that COL12A1 expression was enhanced in patients with high‐grade PAAD (*p* < .05, Figure 5C). Besides, correlation analysis revealed a positive association between PABPC1 and COL12A1 expression in PAAD (*p* < .05, Figure 5D). These outcomes implied that PABPC1 may help mediate COL12A1 expression in PAAD progression.

### 3.5 COL12A1 overexpression reverses the inhibition of BXPC3 cells proliferation and metastasis triggered by PABPC1 silencing

**Figure 6:**
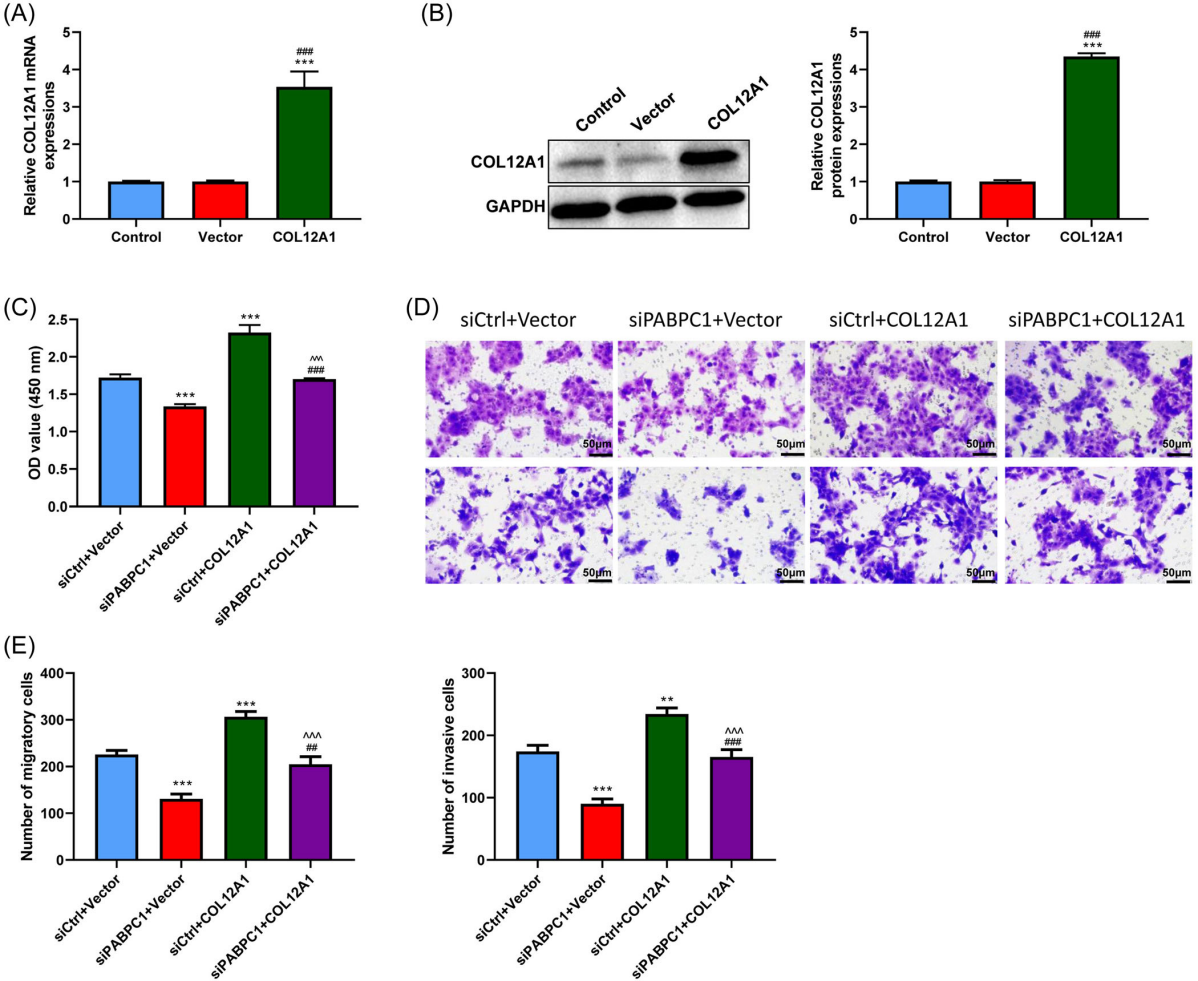
Collagen type XII α1 chain (COL12A1) overexpression reverses the prohibitive effect of cytoplasmic poly (A) binding protein‐1 (PABPC1) on BXPC3 cells growth and metastasis. The pcDNA3.1 vector was adopted for establishment of COL12A1 overexpressed vector. After transfection for 48 h, (A) Reverse transcription‐quantitative polymerase chain reaction was performed to assess the efficiency of transfection. (B) Western blot assay was applied to determine the protein level of COL12A1 in transfected BXPC3 cells. After co‐transfection with PABPC1 silenced vectors (siPABPC1) and COL12A1 overexpressed vectors, (C) Cell Counting Kit‐8 assay was utilized to calculate BXPC3 cells viability. (D and E) Transwell assay was carried out to evaluate the abilities of BXPC3 cells migration and invasion (magnification = ×200, Scale bar = 50 μM). These experiments were repeated for three times and analyzed via SPSS 12.0 software using analysis of variance along with Tukey's post hoc test. **p < .01, ***p < .001 versus Control or negative controls (siCrtl) + Vector; ^##^ *p* < .01, ^###^ *p* < .001 versus Vector or siPABPC1 + Vector, ^^^^^ *p* < .001 versus siCtrl + COL12A1.

Further, to determine whether COL12A1 was involved in modulating PABPC1‐affected growth and metastasis of BXPC3 cells, an overexpressed COL12A1 vector was transfected into BXPC3 cells. RT‐qPCR and Western blot analysis results indicated that COL12A1 expression was upregulated in BXPC3 cells with overexpressed vector transfection compared with that in BXPC3 cells with a blank vector (*p* < .001, Figure 6A,B). As depicted in Figure 6C–E, PABPC1 silencing impeded cell viability, migration, and invasion in BXPC3 cells (*p* < .001). Meanwhile, COL12A1 overexpression clearly reversed the suppression of BXPC3 cell viability, migration, and invasion induced upon PABPC1 silencing (*p* < .01, *p* < .001). These consequences hinted that PABPC1 silencing may inhibit the proliferation and metastasis of BXPC3 cells and affect the progression of PAAD by modulating the expression of COL12A1.

## 4 DISCUSSION

PAAD is a type of gastrointestinal malignancy lacking early screening and diagnosis and has become a global health problem.^21^ The nosogeny of PAAD is a complex process triggered by the interaction of genetic and environmental factors.^22^, ^23^ Elucidating the factors linked to the occurrence and development of PAAD is necessary to further comprehend the etiology and seek novel therapeutic targets. Herein, our research showed that PABPC1 was expressed at high levels in patients with PAAD, which was associated with poor prognosis. RNA‐Seq analysis revealed 474 abnormally expressed genes in BXPC3 cells after transfection with PABPC1 silencing vectors. These DEGs from the top 10 of GO and KEGG analysis were enriched in cell adhesion pathways. Moreover, the silencing of PABPC1 contributed to the regulation of the gene expression profile, hindered cell growth and metastasis, and affected the expression of cell adhesion‐associated genes in BXPC3 cells. Additionally, the PABPC1‐induced upregulation of COL12A1 was observed in BXPC3 cells. The suppressed effects of PABPC1 silencing in BXPC3 cell growth and metastasis were reversed upon COL12A1 overexpression. These consequences indicated that PABPC1 accelerated the progression of PAAD by mediating COL12A1 expression.

With the advent of high‐throughput genetic analysis, gene expression profiling has become an effective method to identify DEGs in various diseases, particularly in cancers.^24^ The human PABPC family has five homologous proteins: PABPC1, PABPC3, PABPC4, PABPC5, and PABPC1L.^25^ PABPC1, the first member of the PABPC family discovered and identified in 1987, is distributed in the cytoplasm and binds to the poly‐A region at the 3′ end of mRNAs to regulate mRNA stability and translation.^26^, ^27^ Based on evidence from the Chinese Glioma Genome Atlas (CGGA) data set, PABPC1 is expressed at the lowest levels in GBM, which was validated by evidence from the TCGA.^28^ By analyzing the gene expression data of PAAD samples in the TCGA database, the outcome indicated that PABPC1 was abnormally expressed in PAAD tissues compared with that in normal pancreatic tissues, and its expression was associated with prognosis.^29^ Similar to the abovementioned findings, the upregulation of PABPC1 was clearly observed in patients with PAAD, which was also connected with the shorter overall survival durations according to GEPIA2 from the TCGA database.

The biological effects of PABPC1 expression in major translation zone aggregation, which can enter the nucleus under biological stress.^30^ Currently, the role of PABPC1 expression is primarily researched in cancer, neurodegenerative diseases, cardiovascular diseases, and spermatogenesis disorders.^12^, ^31^ Chorghade et al. demonstrated that the specific upregulation of PABPC1 in cardiomyocytes could trigger protein synthesis and physiological myocardial hypertrophy.^32^ Additionally, the inhibition of PABPC1 expression has been certified to influence the replication of *Listeria monocytogenes*, thus better modulating inflammatory response.^33^ Although multiple studies have been conducted on PABPC1 expression, the exploration of PABPC1 function in various aspects, especially with respect to immunity and inflammation, remains scarce. Therefore, the mechanism of action of PABPC1 should be studied in greater depth.

It is recognized in the field of cancer research that the expression of cell adhesion‐related genes in cancer cells helps regulate the progression of metastasis.^34^ Using the GO enrichment analysis of genes positively and negatively regulated by PABPC1, PABPC1 was demonstrated to regulate cell adhesion‐related pathways in PAAD cells. Matsuyama et al. reported that microRNA (miR)‐27b inhibited colorectal tumor progression by regulating ARFGEF1 expression and focal adhesion signaling.^35^ Additionally, the IL‐17 signaling pathway was certified to regulate the tumor microenvironment in PAAD.^36^ In our research, KEGG analysis revealed that these DEGs regulated by PABPC1 were enriched in various cancer‐related pathways, which also included the IL‐17 signaling pathway and focal adhesion. These findings hinted that PABPC1 may be involved in regulating the progression of PAAD and a potential prognostic marker.

By regulating the stability and translation of mRNA in the cytoplasm, PABPC proteins play a major role in early embryonic development, cardiomyocyte growth, erythrocyte differentiation, and the induction and development of tumors.^37^, ^38^ Numerous studies have shown that PABPC1 affects multiple biological events, such as tumor cell proliferation, apoptosis, and metastasis, which showcases a key role of PABPC1 in the occurrence and development of tumors.^39^, ^40^ Compelling evidence demonstrates that PABPC1 knockdown suppresses gastric cancer cell proliferation and promotes apoptosis.^41^ The research indicated that PABPC1 induced carcinogenesis in gastric cancer by regulating miR‐34c. Additionally, Feng et al. showed that PABPC1 acted as an oncogene to facilitate ovarian cancer cell growth and invasion, partly by regulating epithelial‐mesenchymal transition (EMT).^11^ Consistent with the abovementioned results, we observed that PABPC1 silencing prohibited cell growth, migration, and invasion in BXPC3 cells, which could be a biomarker for PAAD therapeutics.

Cell adhesion molecules include proteins from the selectin family, integrin family, cadherin family, and some unclassified families such as the CD44 family.^42^ Cell adhesion‐related genes can mediate the interaction between tumor cells and the extracellular matrix, vascular endothelial cells, and parenchymal organ tissue cells and are closely associated with tumor invasion and metastasis.^34^, ^43^ As important cell adhesion‐correlated genes, AZGP1 served as a tumor suppressor in PAAD by inducing EMT.^44^ The upregulation of ARHGAP30 prohibited cell proliferation and metastasis in PAAD cells by inactivating the β‐catenin pathway.^45^ CAV1 was reported to promote glycolysis in PAAD cells and induce malignancy.^46^ COL12A1 overexpression was observably linked to poor prognosis in patients with PAAD.^47^ In our research, we observed that the silencing of PABPC1 upregulated AZGP1 and ARHGAP30 but downregulated CAV1 and COL12A1 expression in BXPC3 cells. Next, we explored the regulatory mechanism of COL12A1 in PABPC1 influenced PAAD progression. Our research indicated that COL12A1 expression was positively correlated with PABPC1 silencing in PAAD, and the upregulation of COL12A1 was associated with poor prognosis in PAAD. Furthermore, COL12A1 overexpression overturned the function of PABPC1 silencing in BXPC3 cell proliferation, migration, and invasion. Thus, the effect of PABPC1 on PAAD may be closely related to COL12A1 expression.

## 5 CONCLUSION

Collectively, our research findings indicated that PABPC1 acts as an oncogene, expediting the proliferation and metastasis of BXPC3 cells by regulating COL12A1 expression. Our findings indicated that PABPC1 is likely a candidate biomarker for PAAD. These findings may lead to the development of a novel method for PAAD treatment; however, in vivo experiments are still necessary to further elucidate the underlying mechanism of action of PABPC1 in PAAD.

## AUTHOR CONTRIBUTIONS

**Weijie Yao**: Writing—original draft. **Yanrong Yao**: Formal analysis. **Wen He**: Formal analysis. **Chengsi Zhao**: Formal analysis. **Di Liu**: Formal analysis. **Genwang Wang**: Formal analysis. **Zuozheng Wang**: Conceptualization; Writing—review and editing.
